# Testosterone Promotes Glioblastoma Cell Proliferation, Migration, and Invasion Through Androgen Receptor Activation

**DOI:** 10.3389/fendo.2019.00016

**Published:** 2019-02-04

**Authors:** Dulce Carolina Rodríguez-Lozano, Ana Gabriela Piña-Medina, Valeria Hansberg-Pastor, Claudia Bello-Alvarez, Ignacio Camacho-Arroyo

**Affiliations:** ^1^Unidad de Investigación en Reproducción Humana, Instituto Nacional de Perinatología-Facultad de Química, Universidad Nacional Autónoma de México (UNAM), Mexico City, Mexico; ^2^Departamento de Biología, Facultad de Química, Universidad Nacional Autónoma de México, Mexico City, Mexico; ^3^Institute for Neurosciences, Hôpital Saint Eloi, INSERM U1051, Montpellier, France

**Keywords:** Glioblastomas, testosterone, androgen receptor, cell proliferation, cell migration, cell invasion

## Abstract

Glioblastomas (GBM) are the most frequent and aggressive human brain tumors due to their high capacity to migrate and invade normal brain tissue. Epidemiological data report that GBM occur in a greater proportion in men than in women (3:2), suggesting the participation of sex hormones in the development of these tumors. It has been reported an increase in testosterone (T) levels in patients with GBM. In addition, androgen receptor (AR) is overexpressed in human GBM, and genetic silencing of AR, and its pharmacological inhibition, induce GBM cell death *in vivo* and *in vitro*. However, the role of T in proliferation, migration and invasion in human GBM cell lines has not been evaluated. We observed that T increased the number of U87, U251, and D54 cells derived from human GBM due to an increase in cell proliferation. This induction was blocked with flutamide, an antagonist of AR. T also induced migration and invasion of GBM cells that flutamide partially blocked. These data suggest that T through AR contributes to the progression of GBM by promoting proliferation, migration, and invasion.

## Introduction

Glioblastomas (GBM) or grade IV astrocytomas are the most aggressive and frequent tumors in the Central Nervous System (CNS). They arise from uncontrolled proliferation of astrocytes, precursor glial cells, and cancer stem cells, and they are generally located in the brain cortex, basal ganglia and thalamus (1). GBM present their highest incidence in humans between 45 and 70 years-old, and the average survival time after diagnosis is around 12–16 months (2). GBM treatment mainly consists of surgical resection, as well as radio and/or chemotherapy. However, due to its infiltration capacity, it is practically impossible to completely extract the tumor, and it relapses (3, 4).

The CNS is an important target for sexual steroids such as androgens (5). Testosterone (T) is the main circulating androgen in men, and in addition to sexual functions, this hormone and its main metabolite, dihydrotestosterone (DHT), regulate diverse functions in the brain such as neuronal differentiation and brain masculinization (6), emotional states, impulsive and aggressive behavior, learning and memory (7, 8). Rapid modulation of dendritic spines induced by hippocampal T and DHT are essential in synaptic plasticity (9). Besides, androgens are involved in the regulation of pathological processes such as tumor growth (10, 11). They can exert their multiple effects through the interaction with its intracellular receptor (AR), a transcription factor that once activated, binds to specific DNA sequences called androgen response elements located in gene promoter regions, thus regulating their expression (12, 13).

It has been found that AR expression was higher in biopsies of GBM patients as compared with that in normal brain tissue (14). Bao et al. (15) observed that AR expression increases according to astrocytomas grade, thus, GBM presented the highest expression. Overexpression of AR has also been observed in several cell lines derived from human GBM (14). In addition, it has been reported that genetic silencing of AR and its pharmacological inhibition induce GBM cell death *in vivo* and *in vitro*, decreasing GBM growth (15, 16).

Besides, it has been reported an increase in testosterone (T) levels in patients with gliomas as compared with patients with a benign tumor or brain injury (15). These data suggest that T-activated AR signaling should play a role in the physiopathology of GBM. This proposal is reinforced by the fact that GBM are more frequent in men than in women in a 3:2 ratio (17). In the present study, we investigated the participation of T and AR activation in GBM cell proliferation, migration, and invasion.

## Materials and Methods

### Cell Culture

U87, U251, and D54 cell lines derived from human GBM were used in this study. U87 and U251 cell lines were acquired at ATCC, and D54 cell line was generously obtained by Dr. Andrés Gutiérrez from Dr. Sontheimer's (University of Alabama, Birmingham, AL, USA). Cell lines were cultured with Dulbecco's Modified Eagle Medium with phenol red (DMEM, Biowest, FRA) and supplemented with fetal bovine serum 10% (FBS, Biowest, FRA), pyruvate (1 mM; InVitro SA, MEX), non-essential amino acids (0.1 mM; InVitro SA, MEX), and a mix of antibiotics (1 mM; InVitro SA, MEX). Cells were incubated with CO_2_ at 5% and at 37°C. Cells were grown until reaching a 70–80% confluence.

### Treatments

Twenty-four hours before treatments cells were grown in phenol red-free DMEM medium (In Vitro S.A., MEX) supplement with FBS (10%) without hormones (charcoal stripped, GeneTex, USA). In order to determine the T concentration that significantly modifies the number of GBM cells, they were treated with testosterone (T, 1, 10, 100 nM and 1 μM in 0.01% ethanol; Sigma, NLD), and vehicle (V, 0.01% ethanol). T (100 nM), competitive antagonist of AR: flutamide (F 5 μM; Sigma, USA), F plus T (F was added 1 h before T), and vehicle were used to evaluate proliferation. T (100 nM), F (10 μM), F plus T, and vehicle were used in migration, invasion and Western blot experiments.

### Cell Counting

1 × 10^4^ U87 cells, and 7 × 10^3^ U251 and D54 cells were seeded in 24-well plate and grown for 24 h. Cells were treated at 0 h of each experiment as described in “Treatments” section. Cells were harvested with 1 mL PBS-EDTA (1 mM) and stained using trypan blue (0.4%) every 24 h during 120 h. In four fields per duplicate, live and dead cells were quantified with a hemocytometer (Neubauer chamber on Olympus BX41, JPN microscope) to test the effect of T and AR activation on cell growth and viability.

### Cell Proliferation Assay

5-bromo-2′-deoxyuridine (BrdU) incorporation assay was used to test the proliferative effect of T. 6 × 10^3^ U87 cells and 4 × 10^3^ U251 and D54 cells were seeded per well in 4-well chamber slides and maintained as described in “Cell culture” section. Cells were treated with T 100 nM at 0 h of each experiment, and BrdU detection with a labeling kit (Roche, DE) was performed according to manufacturer's instructions at 120 h. Fluorescent dye Hoechst 33342 was used to stain DNA. Fluorescence signal was observed at 486 and 515–565 nm with the aid of an Olympus Bx43F fluorescence microscope (Olympus, JPN). Subsequently, F (5 μM) was used to test the role of AR in U87 and D54 cells proliferation at 72 h. The number of cells that incorporated BrdU was quantified with Image J program (NIH, USA), and the percentage of cells positive for BrdU was calculated considering the total number of cells stained with Hoechst.

### Migration Assay

To determine the effect of T on migration of U87, U251, and D54 cells, the “Scratch” test was used to study collective and directional movement of cell populations. 4 × 10^5^ cells were seeded in 6-well plates in DMEM medium and allowed to grow until reaching a confluence of 60–70%, then medium was changed by phenol red-free DMEM, supplemented with FBS (10%) without hormones (charcoal stripped). Twenty-four hours later (when monolayer was absolutely confluent), cells were washed with PBS, then 500 μL of PBS-EDTA (1 mM) were added to each well, and immediately two parallel scratches by well were made with a 200 μL pipette tip. Detached cells were removed by aspiration. One milliliter medium phenol free-red DMEM and without hormones was placed; Cytosine hydrochloride β-D-arabinofuranoside (Ara-C, inhibitor of DNA synthesis, 10 μM; Sigma, USA) was added 1 h before treatments to rule out that changes in the number of migrating cells were due to an increase in proliferation. Without removing the medium, hormonal treatments were added and two photographs per well were taken with an Infinity12C camera coupled to an inverted Olympus CKX41 microscope at 100X magnification of the “Scratch” zone at 0, 3, 6, 12, 24, and 48 h. At 24 h the medium and treatments were refreshed. The number of cells that migrated into the wound was counted using the ImageJ software.

### Invasion Assay

To evaluate the effects of T on U87, U251, and D54 cells invasion, Boyden chamber assay was performed. Transwell inserts (8.0 μm membrane, Corning, USA) were placed in 6-well plate and covered with 1 mL of Matrigel (extracellular matrix gel from Engelbreth-Holm-Swarm; Sigma-Aldrich, USA) previously diluted in FBS and phenol red-free DMEM at 2 mg/mL final concentration. The inserts were incubated at 37°C and 5% CO_2_ for 2 h to allow gelation. 5 × 10^5^ cells were seeded in the insert with 1.5 mL phenol red-free DMEM that included hormone treatments and Ara-C (10 μM). The bottom of the wells was filled with 2 mL phenol red-free DMEM with FBS (10%) as chemoattractant (18). Cells were incubated at 37°C and 5% CO_2_ for 24 h. Matrigel was removed with 3 washes of PBS, and cells trapped in the porous membrane were fixed with paraformaldehyde (4%) and stained with crystal violet (1%). Finally, the insert was allowed to dry and observed under a microscope, five random photographs were taken per insert with an Infinity1-2C camera coupled to an inverted Olympus CKX41 microscope at a 100X magnification. The number of invading cells was quantified in the fields taken at random and the corresponding statistical tests were performed.

### Western Blotting

To determine the effects of T and F on AR protein content in human GBM cells, 5 × 10^5^ U87 cells were seeded in 6-well plates and treated for 24 h as described in “Treatments” section. Cells were lysed with RIPA buffer (50 mM Tris-HCl pH 7.5, 150 mM NaCl, 1% Triton, 0.01% SDS, and ethylene diamine tetra acetic acid 0.5 M EDTA, 1 mL) with a mixture of protease inhibitors (p8340, Sigma-Aldrich, USA) at 4°C, and incubated for 1 h under agitation, centrifuged at 14,000 rpm at 4°C for 5 min, and supernatant was separated for storage at 4°C. Lysates were quantified with the Pierce Protein Assay reagent (Thermo Scientific, IL) in the NanoDrop 2000 spectrophotometer (Thermo Fisher Scientific, USA) at 660 nm. Thirty micrograms of total protein were mixed with Laemmli 2X buffer (100 mM Tris-base pH 6.8, 0.1% bromophenol blue, 20% glycerol, 4% SDS, 10% β-mercaptoethanol), and boiled for 5 min. Proteins were separated by electrophoresis in denaturing 7.5% polyacrylamide gels. Samples were separated at 80 volts for 2 h. Proteins were transferred to nitrocellulose membranes (Millipore, USA) in a semi-humid chamber at 20 mA for 1 h. Membranes were blocked with a solution of Bovine Serum Albumin (2% BSA, InVitro SA., MEX) and 3% milk in TBS-0.1% Tween or 5% milk in TBS-0.1% at 37°C for 2 h or overnight at 4°C. Membranes were incubated with a primary anti-AR antibody at a 1:300 dilution (0.7 μg/mL, rabbit anti-AR polyclonal antibody sc-815, Santa Cruz, USA) or with monoclonal AR (D6F11; 0.08 μg/mL Cell signaling) in 5% milk blocking solution at 4°C overnight. They were then washed with 0.1% TBS-Tween 3 times for 5 min, and incubated at room temperature for 45 min with a peroxidase-conjugated anti-rabbit mouse antibody (IgG-HRP, Santa Cruz sc-2357) in a dilution of 1:7,500 (0.05 μg/mL). To remove the antibody, the membranes were washed with stripping buffer (glycine 0.1 M and SDS 0.5%, pH = 2.5) for 10 min at room temperature and blocked at 37°C for 2 h. Subsequently, they were incubated with primary anti-α-tubulin antibody in a 1:1,000 dilution (0.2 μg/mL) (mouse anti-α tubulin monoclonal antibody, Santa Cruz sc-5286) as loading control. Finally, membranes were washed with TBS-Tween 3 times for 5 min and incubated at room temperature for 45 min with a secondary antibody goat anti-mouse conjugated to peroxidase (IgG-HRP, Santa Cruz sc-2005) in a dilution of 1:5,000 (0.08 μg / mL). Chemiluminescence signals were detected exposing membranes to Kodak Biomax Light Films (Sigma-Aldrich, MO, USA) using peroxidase substrate SuperSignal West Femto Maximum Sensitivity (Thermo Scientific, MA, USA). Blot images were captured using a Canon digital camera and bands were quantified with the ImageJ software (National Institute of Health, WA, USA).

### Statistical Analysis

A one-way ANOVA followed by a Tukey test were performed. GraphPad Prism 7 (GraphPad Software 7, Inc., USA) was used to calculate probability values. *p* ≤ *0.05* was considered statistically significant.

## Results

### T Increases the Number of GBM Cells

**Figure 1:**
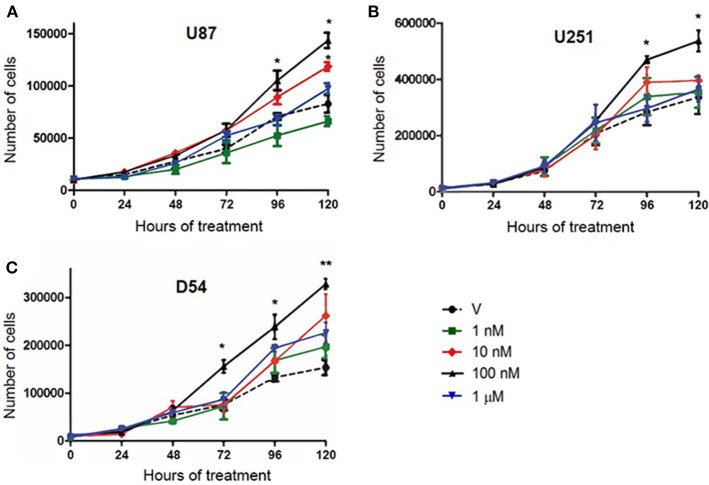
T increases the number of cells derived from human GBM. Number of U87 **(A)**, U251 **(B)**, and D54 **(C)** cells during 120 h of treatment. Each point represents the mean ± SD, *n* = 5. ^*^p ≤ 0.05, ^**^p ≤ 0.01 T vs. V.

To determine whether human GBM cell number is modified by T, we evaluated U87, U251, and D54 cells growth rate through a time course experiment with T at different concentrations (1, 10, 100 nM and 1 μM). We observed a significant increase in the number of cells treated with T 100 nM in the three GBM cell lines from 72 h (D54), and 96 h (U87 and U251) of treatment. No significant difference was observed with T 1 nM and 1 μM (Figure 1). Viability of all cell lines remained constant with all T concentrations throughout the 120 h of treatment with respect to control (Supplementary Figure 1).

### T Effects on the Number of GBM Cells Are Mediated by AR

**Figure 2:**
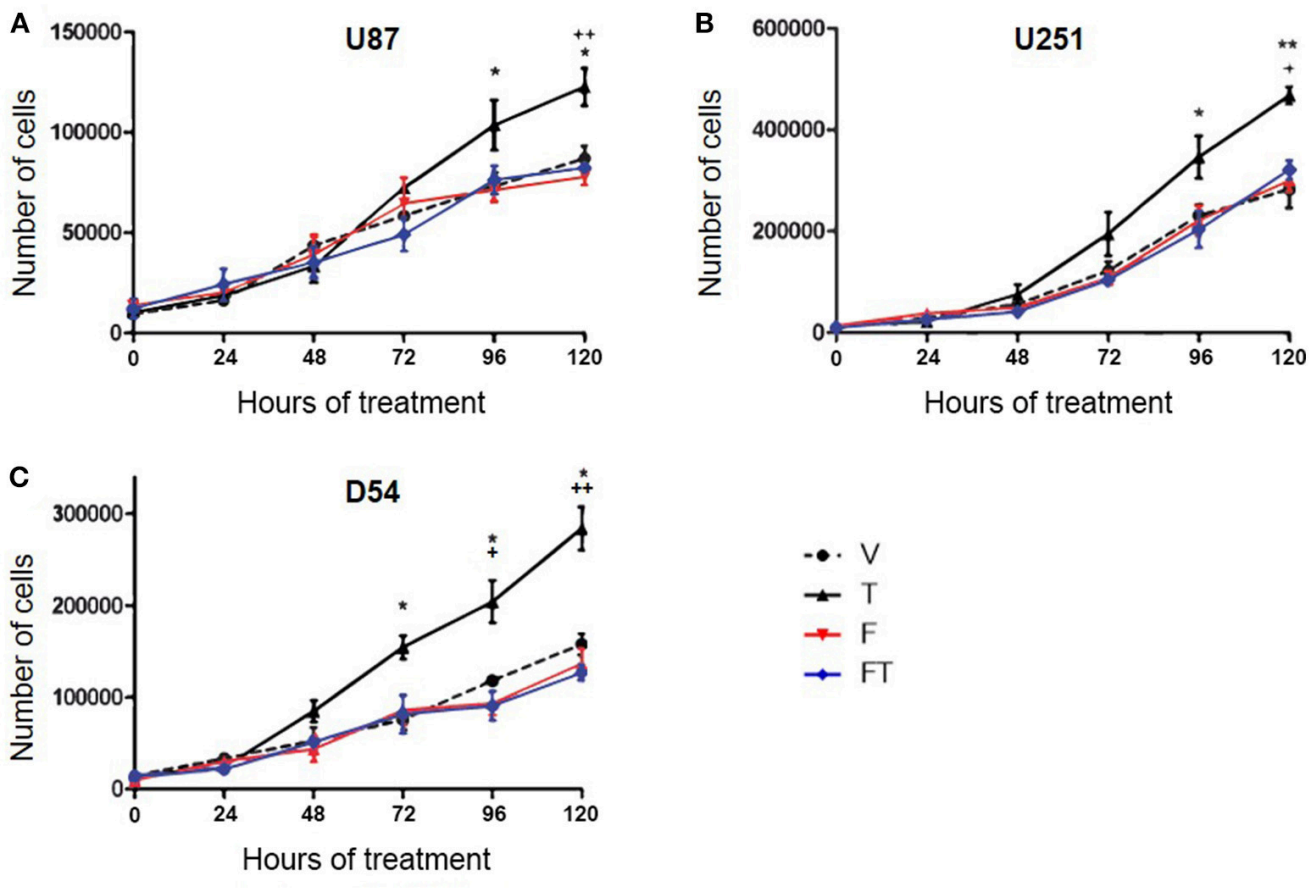
T increases the number of GBM cells through AR. Number of U87 **(A)**, U251 **(B)**, and D54 **(C)** cells during 120 h with vehicle (V), testosterone (T 100 nM), flutamide (F 5 μM), and F plus T (FT). Each point represents the mean ± SD, *n* = 5. ^*^p ≤ 0.05, ^**^p ≤ 0.01: T vs. V; +*p* ≤ 0.05, ++*p* ≤ 0.01 T vs. F and FT.

To determine if AR is involved in the increase in the number of cells induced by T, U87, U251, and D54 cell lines were treated with T (100 nM), competitive antagonist of AR: flutamide (F, 5 μM), F plus T (FT), and vehicle for 120 h. The cell count was carried out for 120 consecutive hours with trypan blue dye. As shown in Figure 1 a significant increase in the number of U87, U251, and D54 cells treated with T (100 nM) was observed. This effect was blocked by F. The single administration of the antagonist did not significantly modify the number of cells (Figure 2). Viability of U87, U251, and D54 cells was not significantly modified with any of the treatments (Supplementary Figure 2).

### Role of AR in U87, U251, and D54 Cell Proliferation

**Figure 3:**
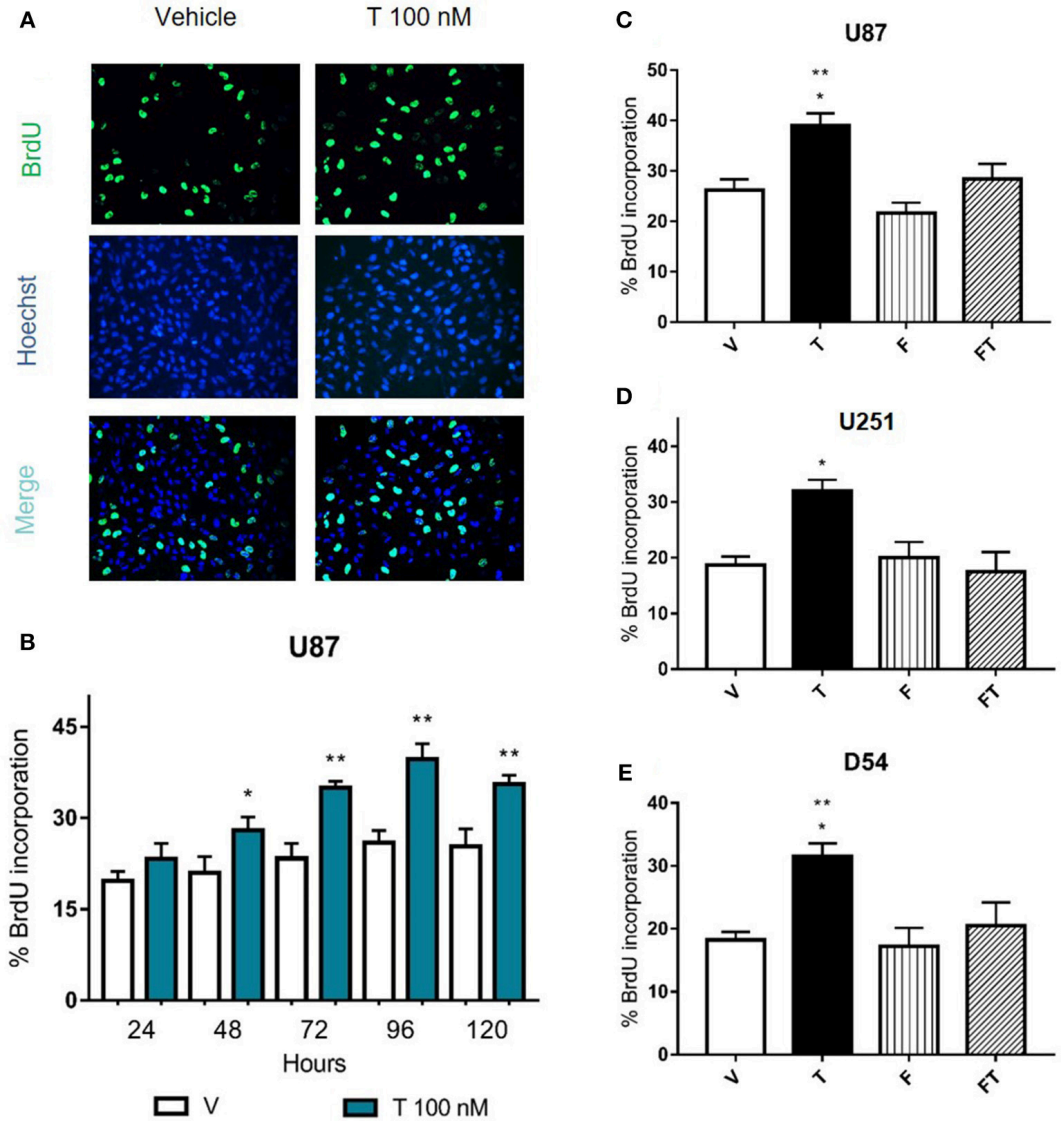
Effects of flutamide on GBM cell proliferation. **(A,B)** Cell proliferation was measured after the treatment of testosterone (T 100 nM) during 24, 48, 72, 96, and 120 h in GBM cells by the BrdU incorporation assay. **(A)** Representative immunofluorescence images (400X magnification) of BrdU-positive U87 cells (upper panel), cell nuclei (Hoechst stain, middle panel), and merge (lower panel) are shown. **(B)** Graph represents the percentage of U87 cells incorporating BrdU. Each bar indicates the mean ± SD, *n* = 4. ^*^p ≤ 0.05, ^**^p ≤ 0.01 T vs. vehicle (V). **(C–E)** AR antagonist flutamide (F 5 μM) blocks the increase in cell proliferation induced by T. Graphs show cell proliferation of U87 **(C)**, U251 **(D)**, and D54 **(E)** cells treated 78 h with V, T, F, and F plus T (FT). Each bar indicates the mean ± SD, *n* = 4. ^*^p ≤ 0.05 vs FT; ^**^p ≤ 0.01 vs V and F.

In order to know if the increase in GBM cell number induced by T is caused by changes in cell proliferation, 5-bromo-2′-deoxyuridine (BrdU) assay was performed at 24, 48, 72, 96, and 120 h in U87 cells. Figures 3A,B shows that T (100 nM) increased the percentage of cells that incorporated BrdU from 48 to 120 h, suggesting that the increase in number of cells is due to proliferation. To determine if T effects on proliferation are mediated by AR, U87, U251, and D54 cells were treated with antagonist F, and F plus T. Data showed that F (5 μM) blocked the proliferative effect of T, while the single administration of F did not modify cell proliferation (Figures 3C–E).

### Role of T in Cell Migration

**Figure 4:**
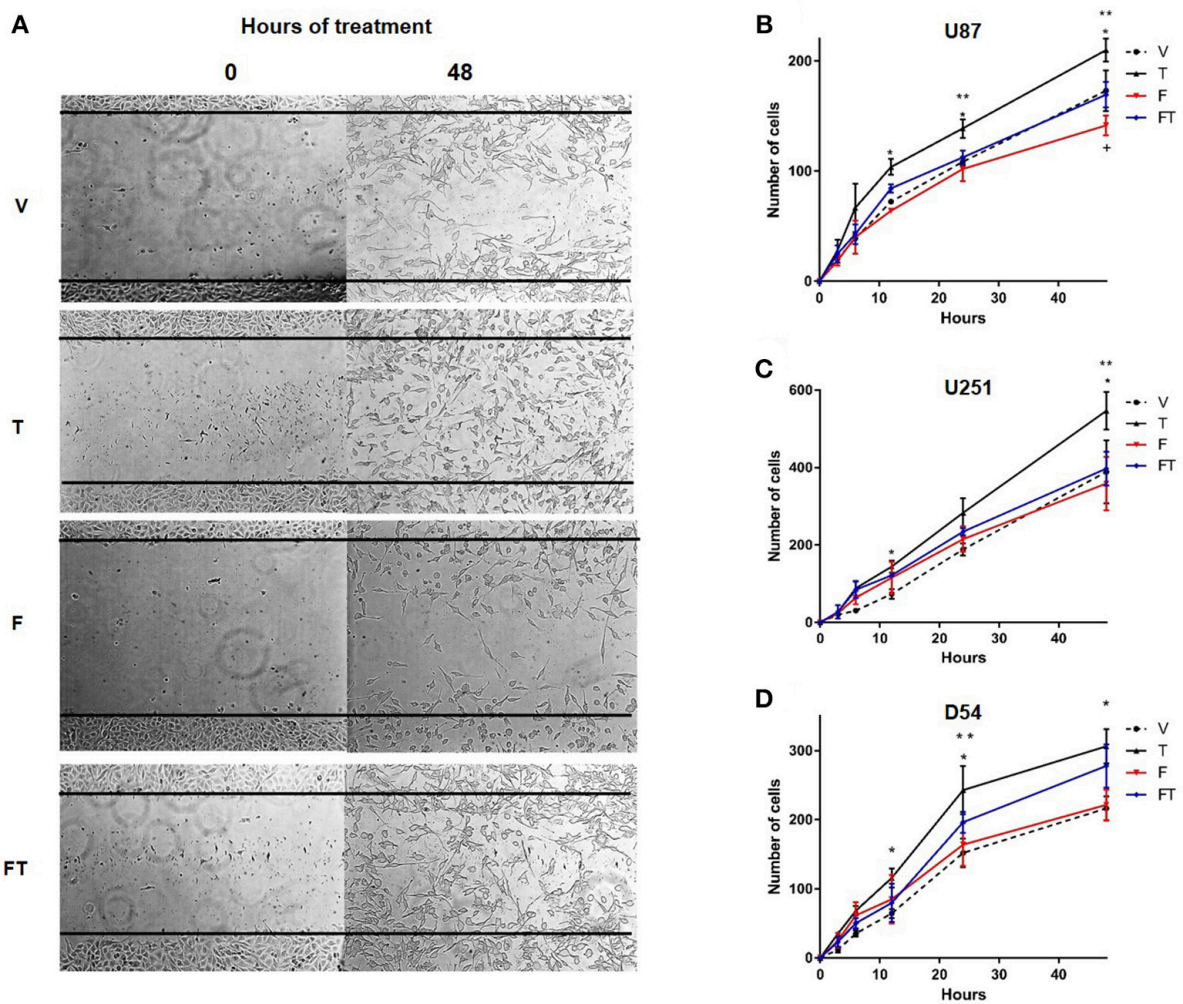
T increases GBM cell migration. **(A)** Representative photographs at 0 and 48 h of the Scratch in D54 cells. Graphs of U87 **(B)**, U251 **(C)**, and D54 cells **(D)** show the number of cells that migrate at 0, 3, 6, 12, 24, and 48 h treated with vehicle (V), testosterone (T 100 nM), flutamide (F 10 μM), and F plus T (FT), each point represents the mean ± SD, *n* = 3. ^*^p ≤ 0.05 T vs V and F; ^**^p ≤ 0.01 T vs FT; +*p* ≤ 0.05 V.

In order to evaluate the effects of T on GBM cell migration, Scratch assays were performed. It was observed that T (100 nM) increased the number of migrating cells with respect to vehicle from 12 to 48 h in U87 and D54 cells, and at 12 and 48 h in U251 cells. F (10 μM) completely blocked T effects in U87 and U251 cells, but only partially in D54 cells. Treatment with a single administration of F had no effect on migration of D54 and U251 cells at the times evaluated as compared with vehicle, but decreased it in U87 cells (Figure 4 and Supplementary Figure 3).

### Role of T in Cell Invasion

**Figure 5:**
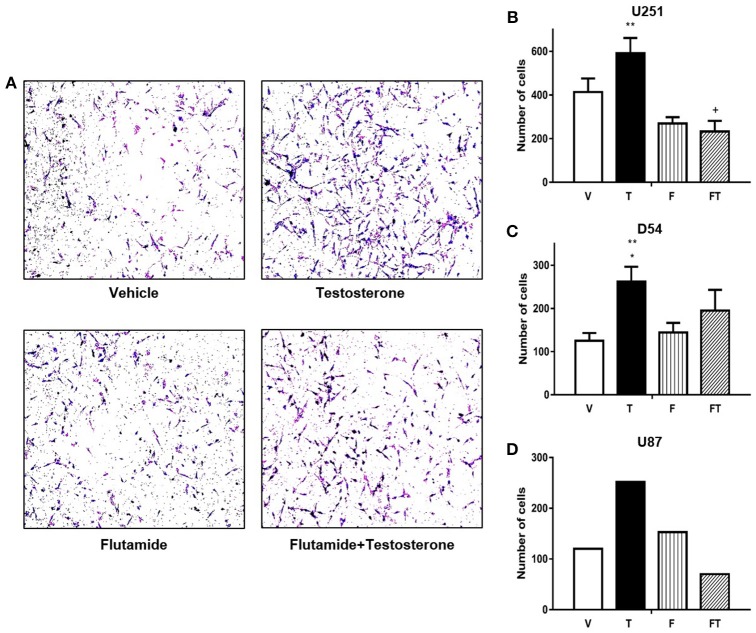
T increases GBM cell invasion. **(A)** Representative photographs are observed in a Boyden chamber assay at 24 h with different treatments in D54 cells. Graphs of U251 **(B)**, D54 **(C)**, and U87 cells **(D)** represent number of cells that invade at 24 h with vehicle (V), testosterone (T 100 nM), flutamide (F 10 μM), and F plus T (TF). Each point represents mean ± SD, *n* = 3 (U251 and D54 cells) and *n* = 1 (U87 cells). ^*^p ≤ 0.05 vs V and F; ^**^p ≤ 0.01 vs FT; + *p* ≤ FT vs V.

The effect of T on cell invasion was evaluated by a Boyden chambers assay. It was found that T (100 nM) increased the number of invasive U251, D54, and U87 cells as compared to vehicle at 24 h (Figure 5 and Supplementary Figure 4). T increased the number of invasive U251 and D54 cells with respect to F (10 μM). F decreased U251 cell invasion induced by T, but it only partially blocked the invasive effect of T in D54 cells (Figure 5).

### T Reduces AR Content in U87 Cells

**Figure 6:**
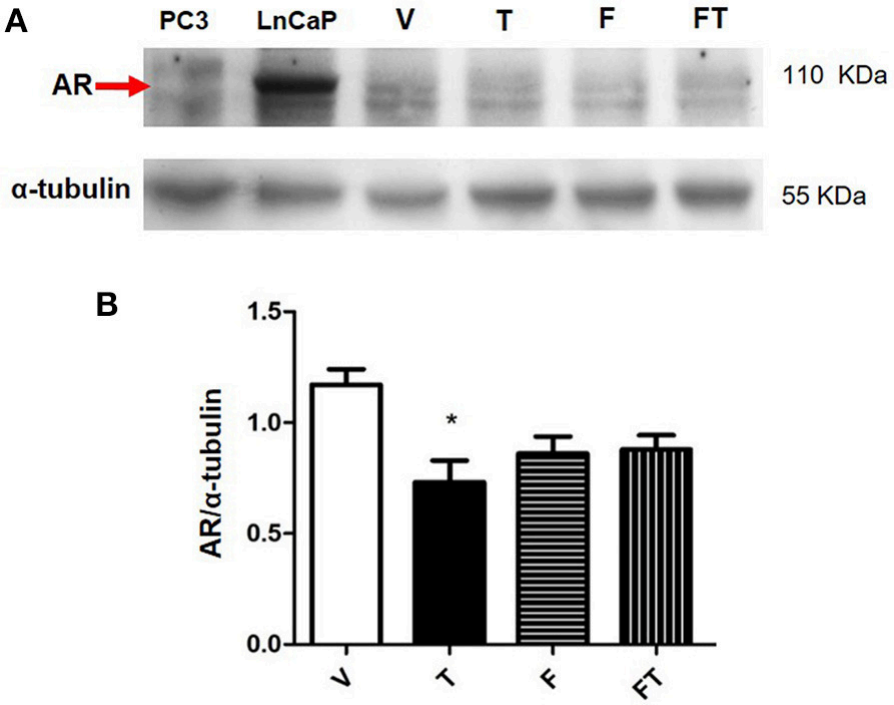
Expression of AR in U87 cells. **(A)** Results obtained from Western blot of PC3 (negative control), LnCap (positive control), and U87 cells treated with vehicle (V), testosterone (T 100 nM), flutamide (F 10 μM), and F plus T (FT), during 24 h. **(B)** Graph represents densitometric analysis of the experiments. Each bar indicates the mean ± SD, *n* = 4. ^*^p < 0.05, T vs. V.

We observed that AR was expressed in the three GBM cell lines used in this study (Supplementary Figure 5). In order to study the regulation of AR expression by T, Western blots were performed. The LnCaP and PC3 cells, both derived from prostate cancer, were used as positive and negative controls, respectively. Figure 6 shows that AR content (determined with the use of polyclonal antibody SC-5286) is lower in U87 cells treated with T (100 nM) during 24 h than in cells treated with vehicle. Treatments with F (10 μM) and F plus T did not show significant differences as compared with vehicle and T. Interestingly, T effect was not clearly observed when AR was detected with the monoclonal antibody D6F11 (Supplementary Figure 5).

## Discussion

The participation of androgens in several physiological and pathological processes in the CNS has been widely described (5). It is also known that they are involved in prostate, colon and lung cancer growth (11, 19–21). Epidemiological data report that GBM occur in a greater proportion in men than in women (3:2), which suggests androgens participation in the development of these tumors. Different evidences suggest that sex hormones can modulate proliferation, migration and invasion of GBM (22–25). Thus, in this work we evaluated the role of T in the progression of GBM.

According to our results, T (100 nM) induces the growth of human GBM cell lines used in this study (U87, U251, and D54), without modifying the percentage of viability. In order to know that cell growth was due to an increase in proliferation, BrdU assay was performed in the three cell lines. It was found that T increased cell proliferation at 48 h and the increase in the number of cells was reflected until 96 h. This difference could be due to the fact that the replication rate of the U87 cell line is 36 h. These data agree with those reported by Merritt and Foran (26) who observed an increase in cell viability with T (1 μM) in T98G at 120 h of treatment. These data suggest that T contributes to the progression of GBM by promoting cell proliferation. Although the inductor effect of T on proliferation was observed at higher concentrations than physiological ones, it has been shown that T levels of patients with some type of astrocytoma are higher than those with some other benign tumor or brain injury (15).

Since GBM present a high infiltration capacity that involves processes of migration and invasion of surrounding tissues of the CNS (27), we evaluated for the first-time motility of GBM cells in response to T. Unlike migration, cell invasion also involves cell adhesion and extracellular matrix degradation, allowing cells to penetrate through tissue barriers such as the basement membrane or stroma (28, 29). Our results indicate that T (100 nM) increased the migration of U87, U251, and D54 cells. It is noteworthy that the increase in number of cells that migrated with T treatment was only due to a greater motility and not to an increase in cell proliferation since experiments were performed in the presence of AraC, a potent inhibitor of α, β, and δ DNA polymerases, which interferes with elongation, during replication and chain repair. Although the half-life of Ara-C is <1 h in most cell lines, more than 80% of Ara-C remains in the DNA up to 24 h (30, 31). F blocked T effects in U87 and U251 cells, but this blockade was only partial in D54 cells, revealing the participation of other mechanisms that could regulate the activity of T, such as the membrane androgens receptor, whose action by the non-classical mechanism has recently been described in prostate cancer and Sertolli cells (32). It has been described that non-genomic androgen actions regulate proliferative/migratory signaling in stromal cells (33), steroid signaling activation and intracellular localization of sex steroid receptors (34).

Similar to the effects observed in migration, T increased invasion in GBM cell lines. As mentioned, infiltration of GBM to areas of healthy brain tissue not only involves migration, but also involves the action of proteins such as metalloproteinases (MMP), therefore, it is possible that T has an effect on some MMP described in GBM (35, 36). Participation of MMPs in motility of GBM cells has been described, as well as a differentiated expression of MMPs among cell lines, which could explain the different results in U251, U87, and D54 cell invasion with T and F (37). As in the case of migration, we observed that the inductor effect of the T was completely blocked by F in U251 cells, but only partially blocked in D54 cells. We cannot rule out the participation of a non-nuclear mechanism of T or its metabolites that could mediate its effect on migration and invasion of GBM cells. Some studies have reported that aromatase (enzyme that synthesizes estradiol from T) is overexpressed in astrocytoma biopsies, besides it has been negatively correlated with the survival of patients, and positively with estradiol concentration (38). Our group previously demonstrated that estradiol increases cellular growth of GBM cells by activation of ERα (39). These data suggest that depending on the status of aromatase the effect should be mediated by T or estradiol. Therefore, the characterization of the expression and activity of this enzyme is fundamental.

Effects of T can be mediated by AR, a nuclear transcription factor member of the family of steroid receptors. It has been reported that AR expression is higher in patients with GBM as compared with normal brain tissue from the same patients (14), and that the expression of AR increases as the degree of malignancy of astrocytomas progresses, being grade IV (GBM) the ones that present the highest protein content (15). Overexpression of AR has also been described in 8 cell lines derived from GBM, including U87 and U251 (15). These data were replicated in our study by Western blot. We evaluated whether the effect of T can be mediated by the AR, using F, a non-steroidal antiandrogen without androgenic properties that it is suitable for using in the treatment of prostate cancer (40, 41). It was observed that F treatment blocks the effect of T on growth of the three cell lines used in this study, as well as the increase in cell proliferation induced by T at 72 h of treatment. It is important to mention that in the cell count and proliferation experiments, a lower concentration of antagonist was used because F 10 μM generated a decrease in cell viability from 72 h. Besides, recent studies have shown that genetic silencing of AR in cell lines and pharmacological inhibition of AR reduces GBM cell growth, and induces GBM cell death *in vivo* and *in vitro* (15, 16).

AR was expressed in GBM cell lines used in the present study. We analyzed the effects of T on AR protein content in U87 cell line with two different antibodies. Although we observed a reduction in AR content when we used the polyclonal antibody SC-5286, the experiments with the monoclonal antibody D6F11 did not demonstrate such reduction. Therefore, additional experiments are required to determine the effect of T in the regulation of AR in GBM cells.

In conclusion, in this work we demonstrated that T induces proliferation, migration, and invasion of human GBM cells through the interaction with AR.

## Data Availability Statement

The raw data supporting the conclusions of this manuscript will be made available by the authors, without undue reservation, to any qualified researcher.

## Author Contributions

DR-L and IC-A conceived the study and wrote the paper. DR-L and CB-A performed and analyzed the experiments. AP-M and VH-P participated in the experimental design, provided technical assistance and contributed to the preparation of figures. All authors reviewed the results and approved the final version of the manuscript.

### Conflict of Interest Statement

The authors declare that the research was conducted in the absence of any commercial or financial relationships that could be construed as a potential conflict of interest.
