# Supplementary material for: Testosterone Promotes Glioblastoma Cell Proliferation, Migration, and Invasion Through Androgen Receptor Activation
Source: Front Endocrinol (Lausanne). 2019 Feb 4;10:16. doi: 10.3389/fendo.2019.00016 (PMC6369181; doi:10.3389/fendo.2019.00016)
Supplement: Supplementary file 1 [file Data_Sheet_1.docx]

Supplementary Material

Testosterone promotes glioblastoma cell proliferation, migration and invasion through androgen receptor activation.

**Dulce Carolina Rodríguez-Lozano^1^, Ana Gabriela Piña-Medina^2^, Valeria Hansberg-Pastor^2^, Claudia Bello-Alvarez^1^, Ignacio Camacho-Arroyo^1*^**

*** Correspondence:** Dr. Ignacio Camacho-Arroyo: [camachoarroyo@gmail.com](mailto:camachoarroyo@gmail.com)

# Supplementary Figures.

Supplementary Figure 1. Effect of testosterone on viability GBM cells. Percentage of viability U87 (a), U251 (b) and D54 (c) cells with different concentrations of testosterone (T) during 5 days. Each point represents the mean ± SD. n = 5.


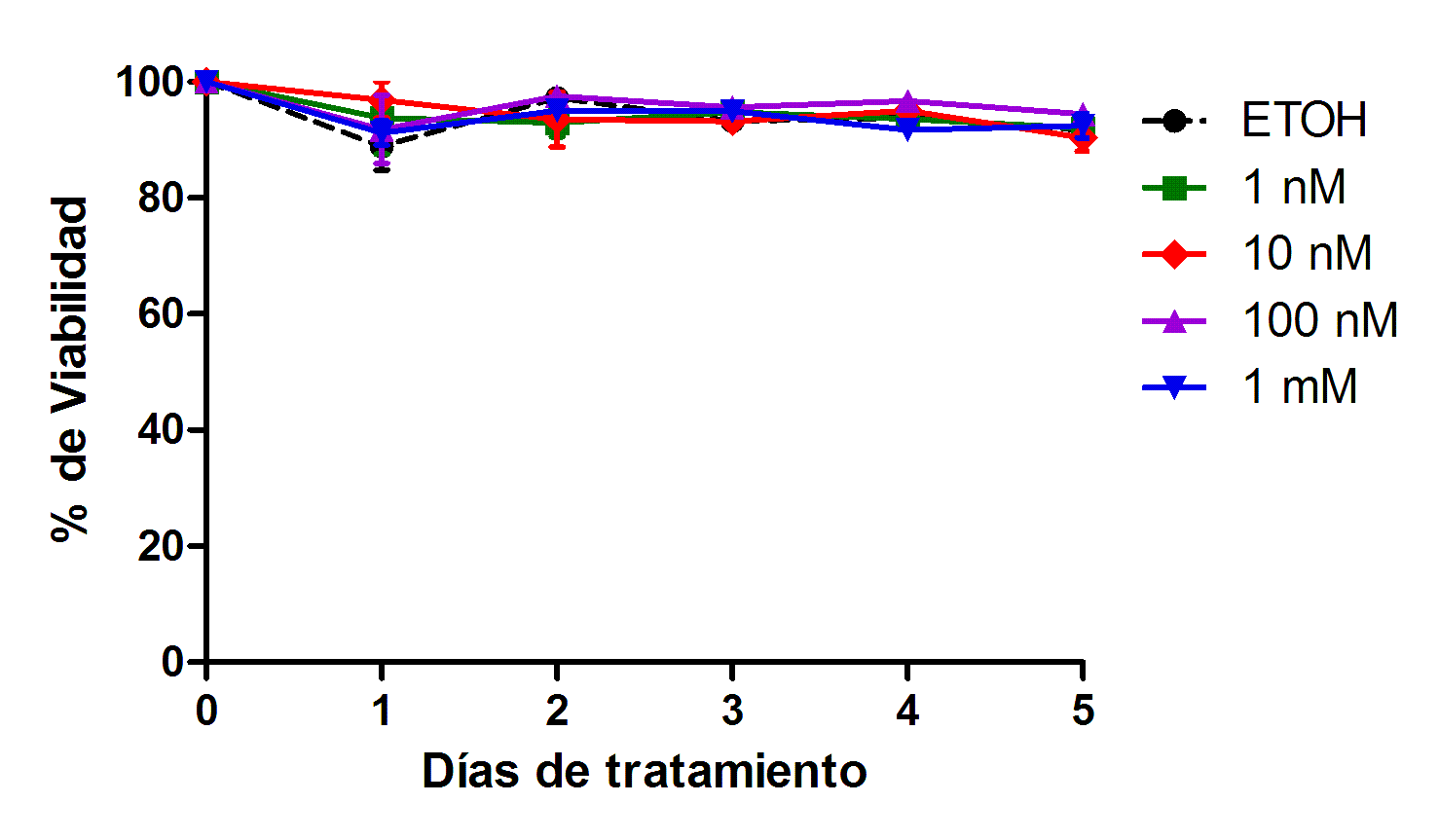

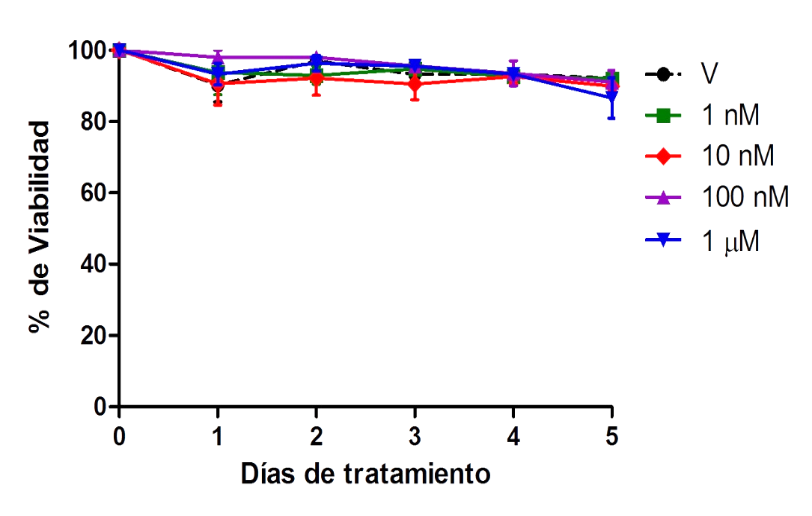

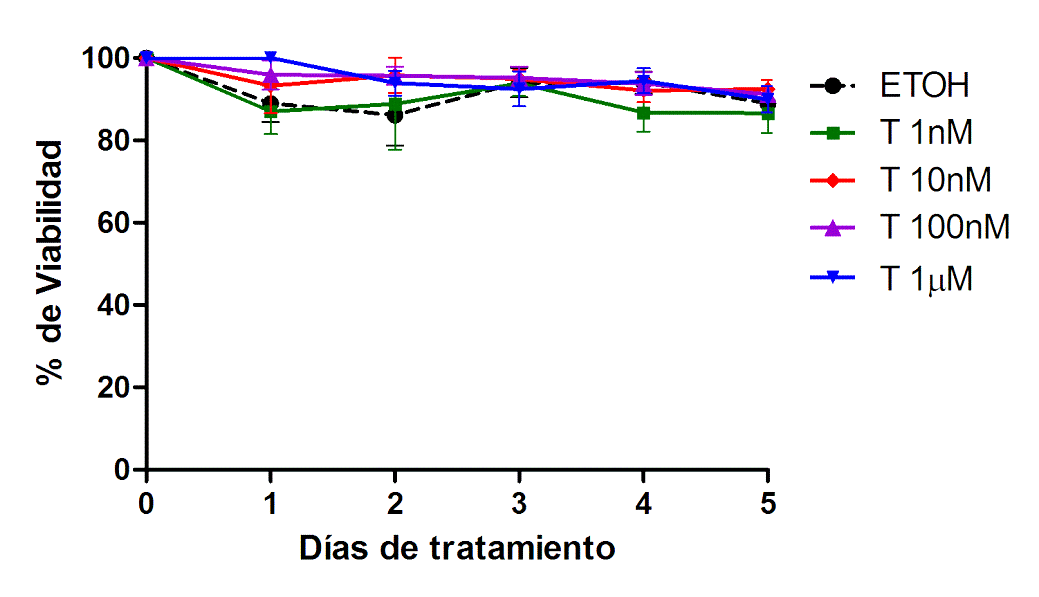

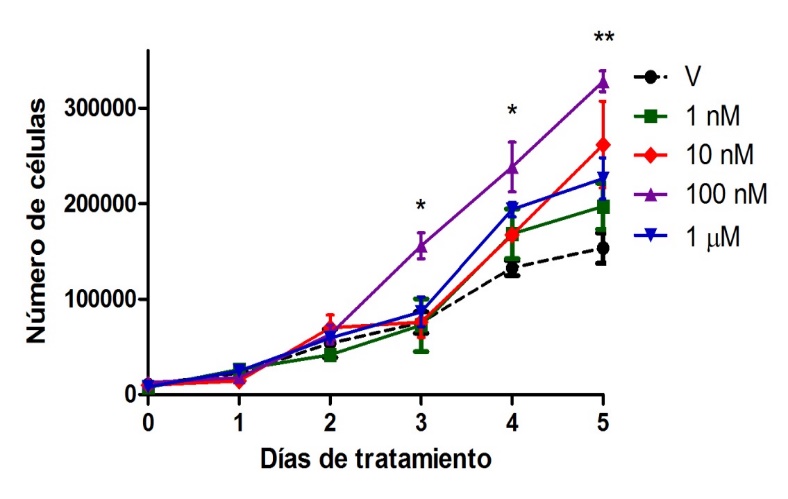


**U87**

**a)**

% Viability

Days of treatment

**U251**

**b)**

% Viability

Days of treatment


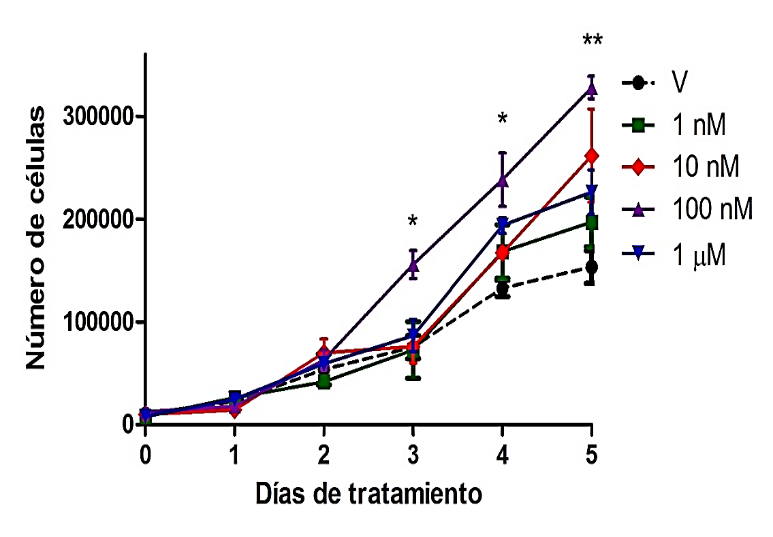


**D54**

**c)**

% Viability

Days of treatment

**Supplementary Figure 2.** **Effect of flutamide on the viability of cells derived from GBM.** Percentage of viability of U87 (a), U251 (b) and D54 (c) cells for 5 days with vehicle (V), testosterone (T 100 nM), flutamide (5 µM) and F + T. Each point represents the mean ± SD., n = 5.


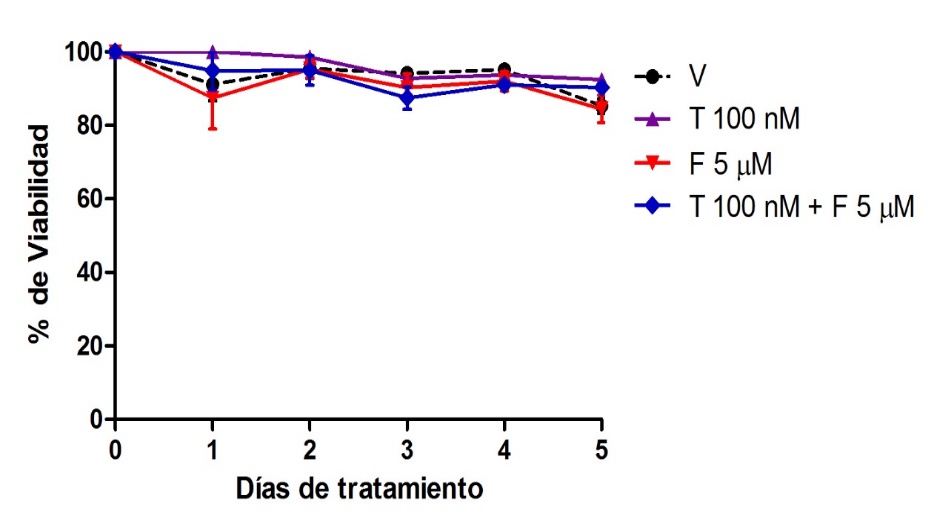

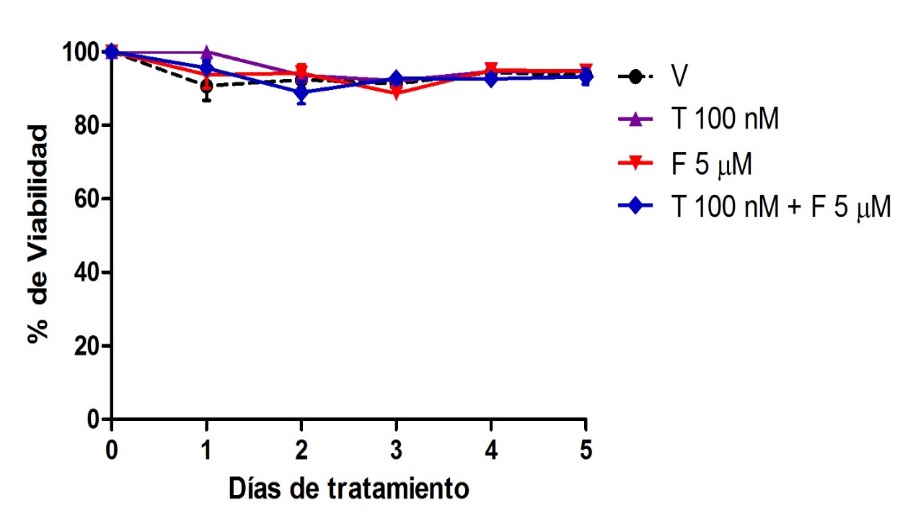

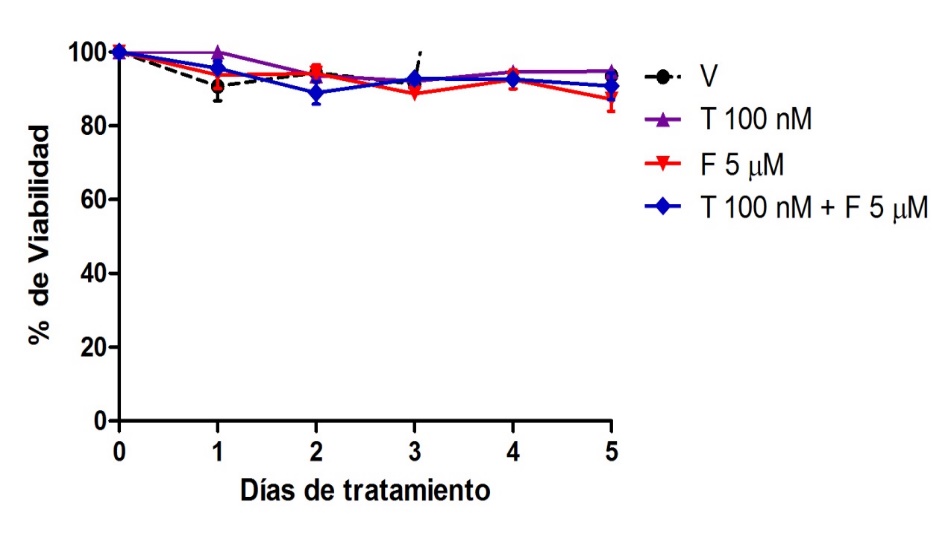


**a)**

**U87**

**U251**

**b)**

**c)**

**D54**


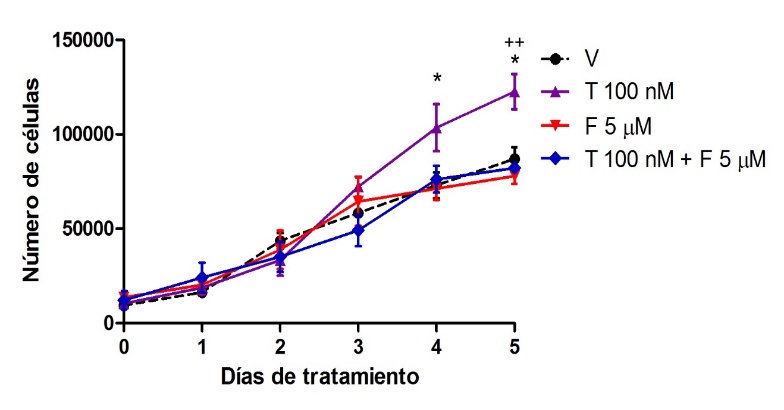


V

T

F

FT

% Viability

% Viability

% Viability

Days of treatment

Days of treatment

Days of treatment

**Supplementary Figure 3.** **Effect of T on the migration of D54 cells.** T increases migration in D54 cells derived from human GBM. Representative photographs at 0, 3, 6, 12, 24 and 48 hours treated with vehicle (V), testosterone (T 100 nM), flutamide (F 10 μM) and F plus T (FT).


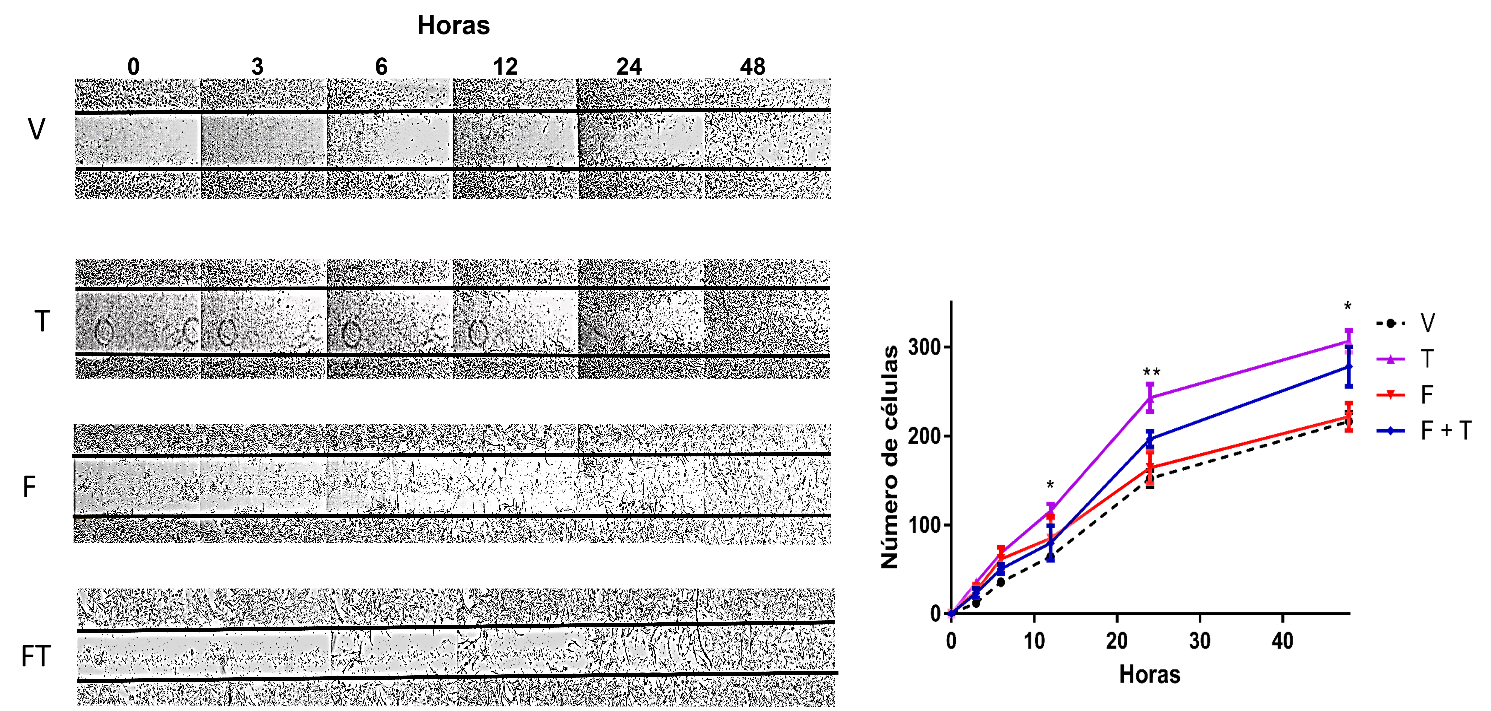


**V**

**T**

**FT**

**F**

**Hours of treatment**


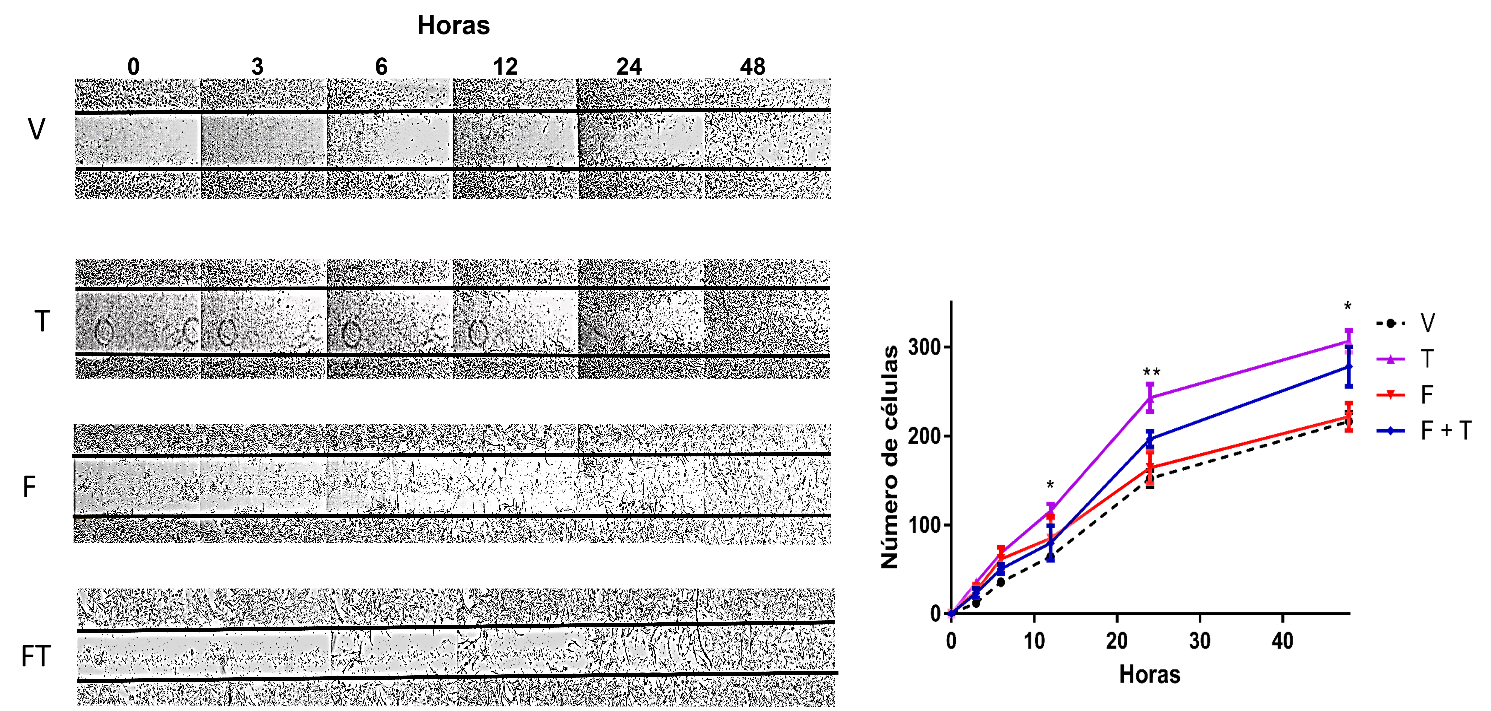

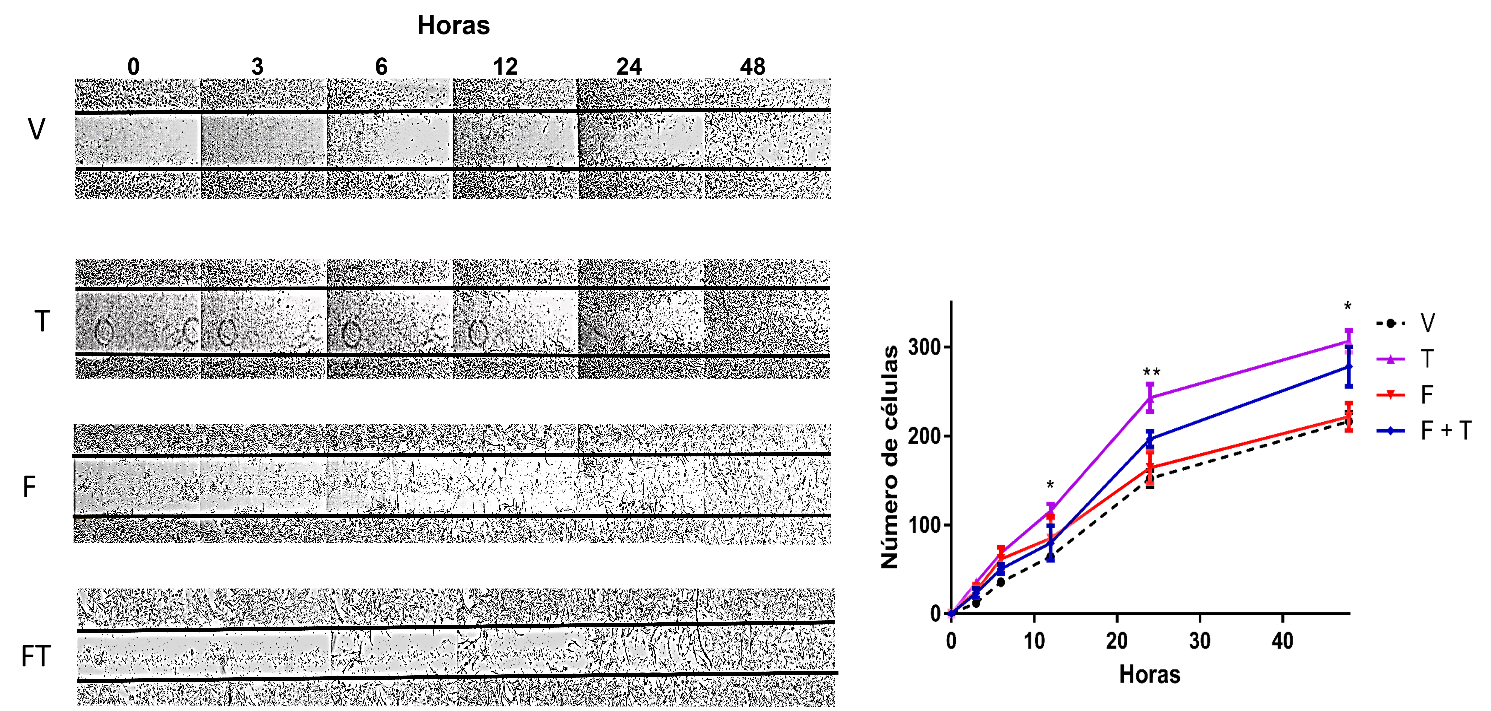

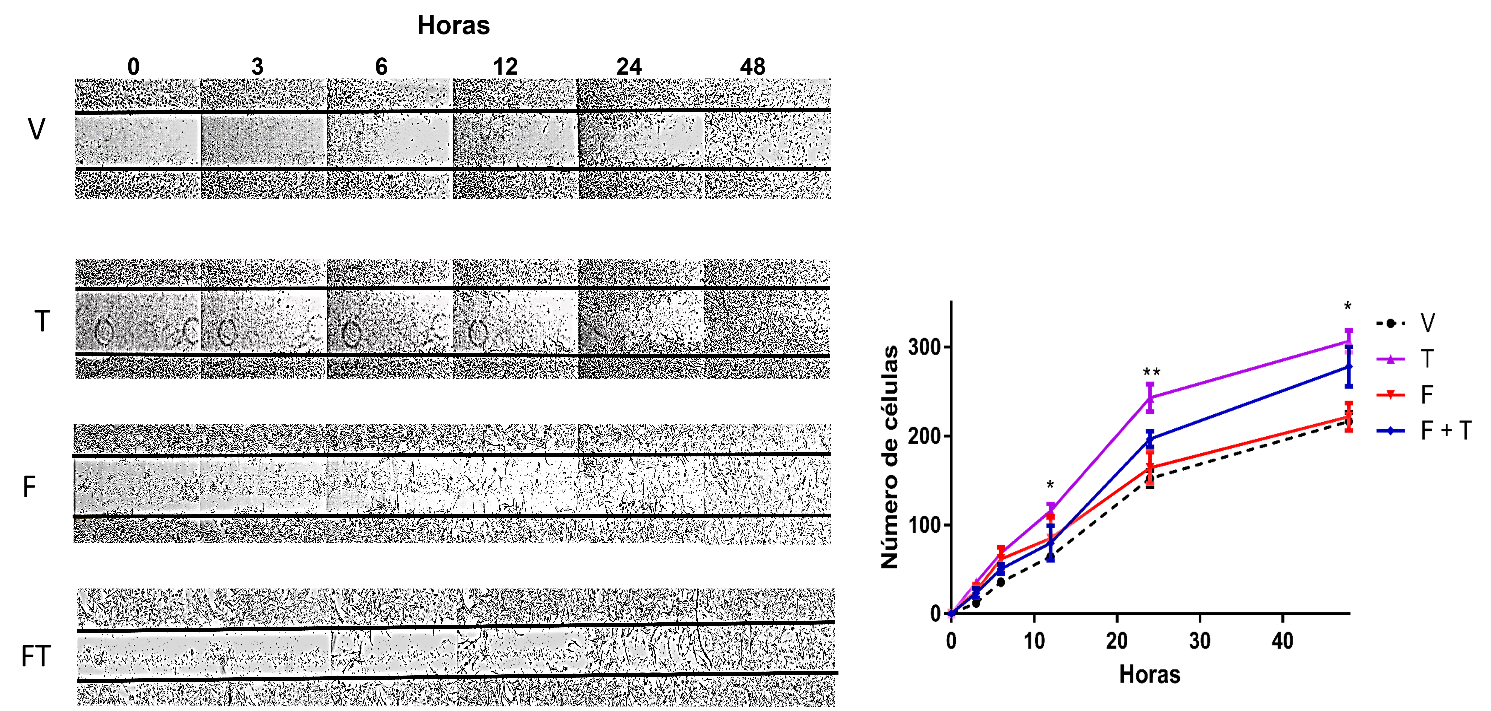


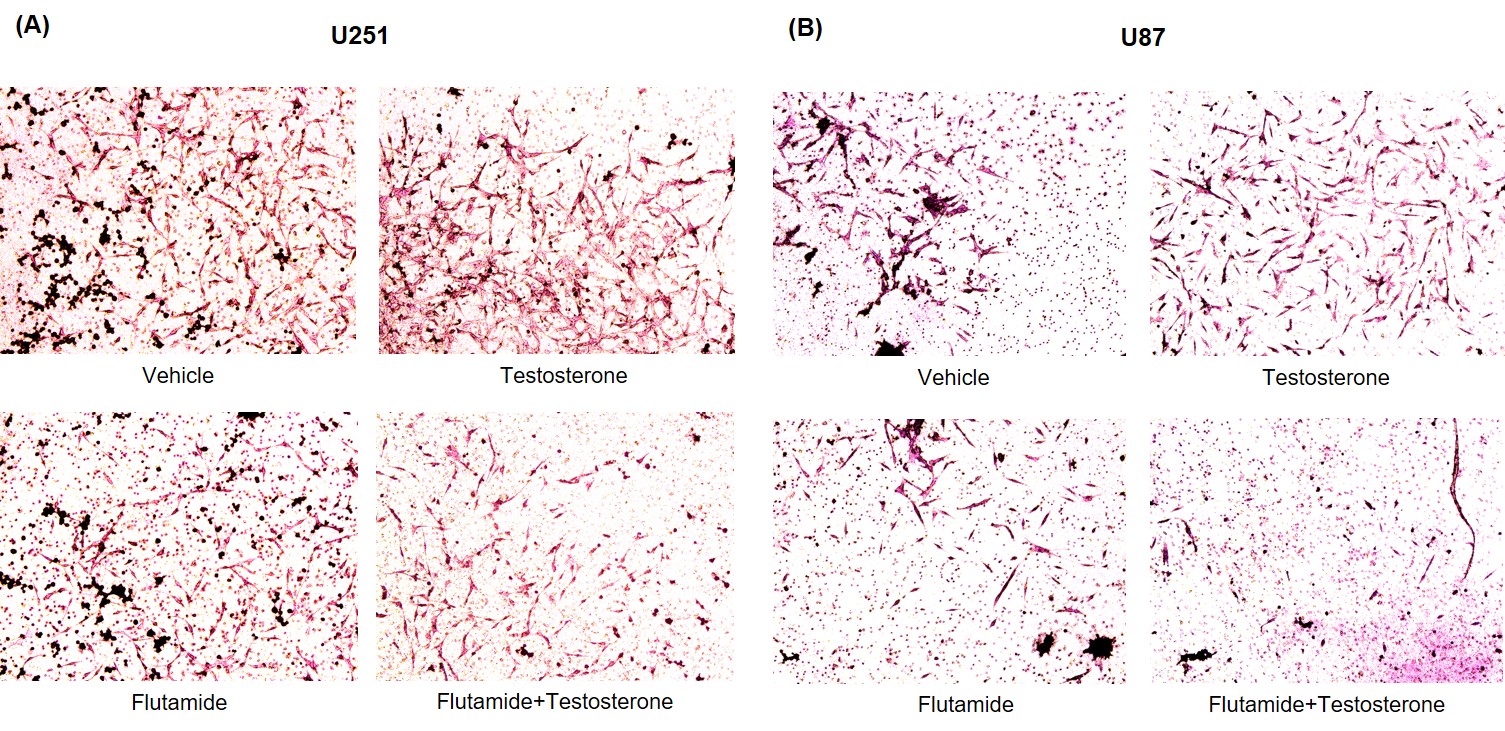


**Supplementary Figure 4.** **Effect of T on the invasion of U251 and U87 cells.** T increases cell ivassion of U251 (A) and U87 (B) cells. Representative photographs are observed in Boyden chamber assay at 24 hours with different treatments: (V), testosterone (T 100 nM), flutamide (10 µM) and F + T.


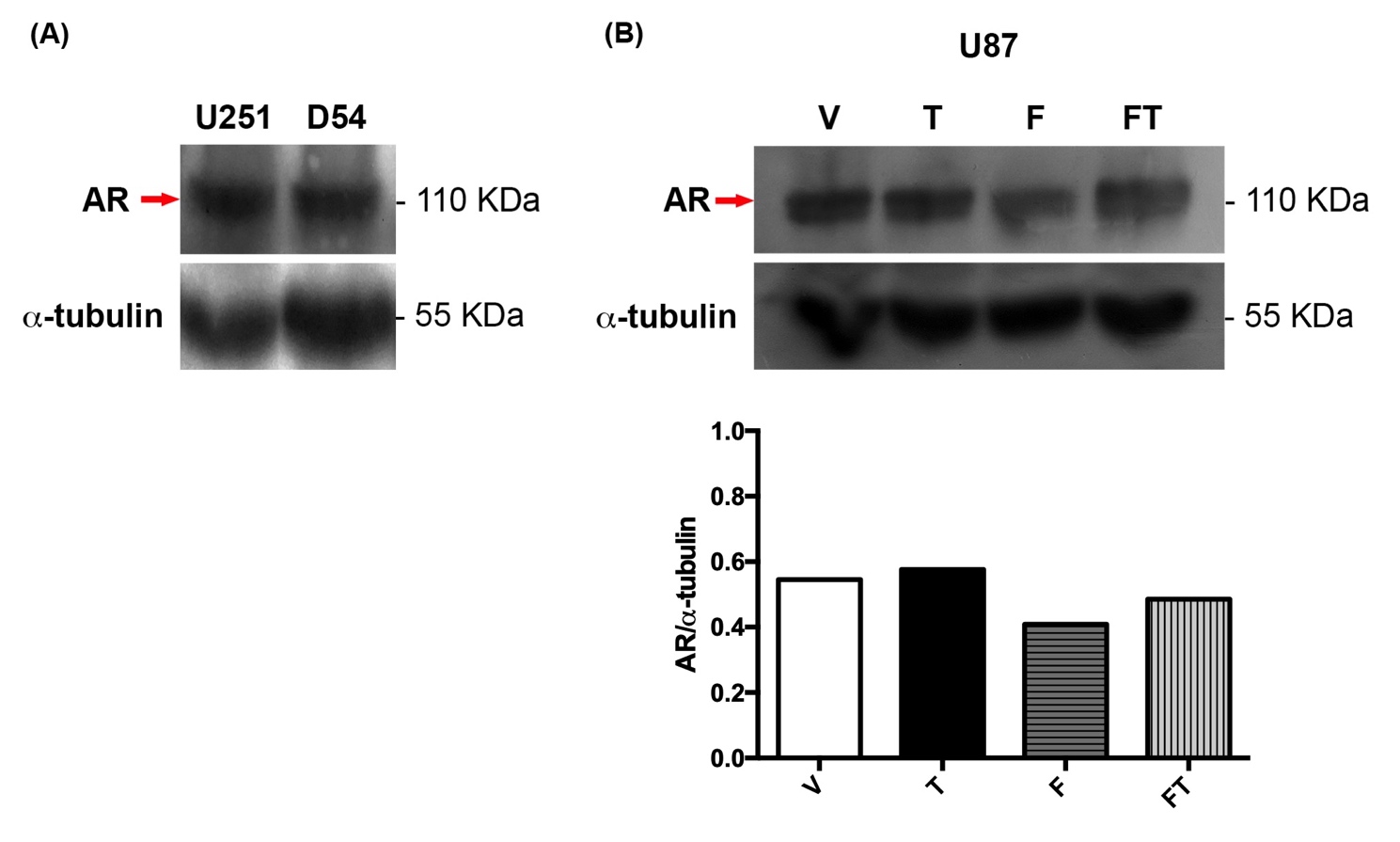
**Supplementary Figure 5.** **Expression of AR in GBM. (A) Basal expresion of AR in U251 and D54 cell lines. (B)** Results obtained from Western blot of U87 cells treated with vehicle (V), testosterone (T 100 nM), flutamide (F 10 μM) and F+T (FT), during 24 hours. AR was detected with monoclonal antibody (D6F11, Cell signaling). Graph represents densitometric analysis of the experiments.
